# Supplementary figures and images for: Two transmembrane transcriptional regulators coordinate to activate chitin-induced natural transformation in Vibrio cholerae
Source: PLoS Genet. 2025 Feb 18;21(2):e1011606. doi: 10.1371/journal.pgen.1011606 (PMC11856585; doi:10.1371/journal.pgen.1011606)

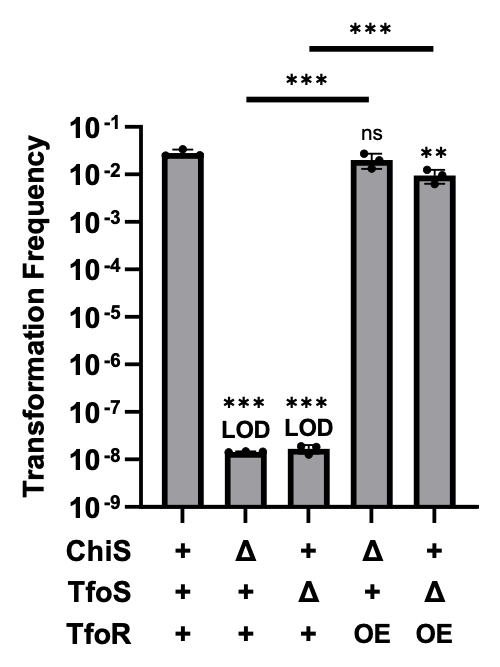

Supplement: S1 Fig — Chitin-dependent transformation assays of the indicated strains. For ChiS genotypes, “+” denotes that cells have a WT copy of ChiS and “Δ” denotes that cells lack ChiS. For TfoS genotypes, “+” denotes TfoSWT and “Δ” denotes that TfoS is deleted. For tfoR, “+” denotes WT tfoR and “OE” denotes that tfoR is overexpressed (Ptac-tfoR + 1µM IPTG). Results are from three independent biological replicates and shown as mean ± SD. Statistical comparisons are made by one-way ANOVA with Tukey’s multiple comparison test on the log-transformed data (normal distribution confirmed by Shapiro-Wilk test). Statistical identifiers directly above bars represent comparisons to the parent. ns, not significant. *** = p < 0.001, ** = p < 0.01. LOD, limit of detection. (TIFF) [file pgen.1011606.s001.tiff]

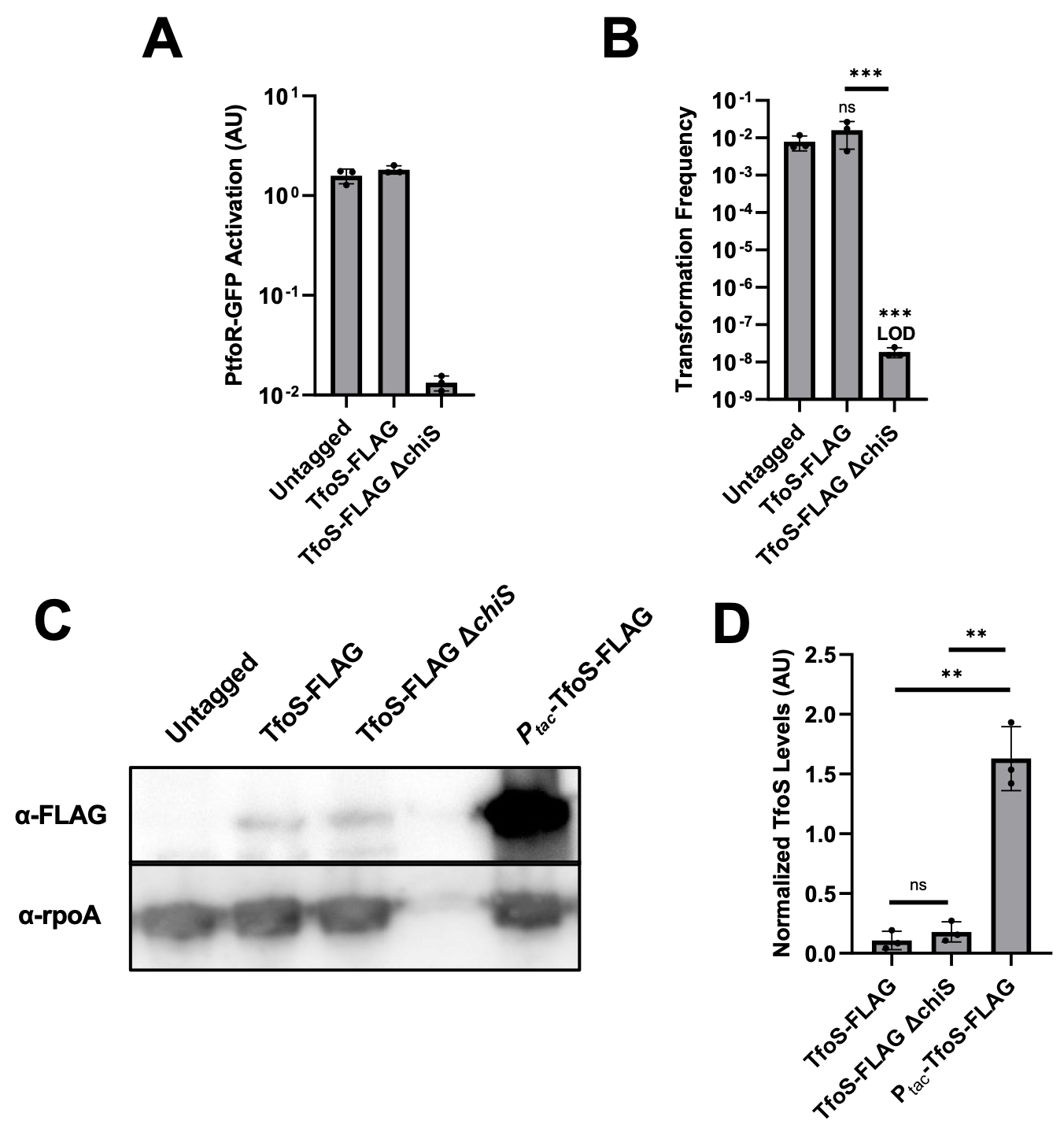

Supplement: S2 Fig — (A) Transcriptional reporter assays to assess chitin-dependent gene expression in the indicated strains. All strains harbored PtfoR-gfp and Pconst2-mTFP1 constructs. Cells were incubated on chitin and then imaged via epifluorescence microscopy to assess reporter expression. For each replicate (n = 3), the geometric mean fluorescence was determined by analyzing 300 individual cells. (B) Chitin-dependent transformation assays of indicated strains. (C) Western blot analysis of the indicated strains. The blot in C is representative of three biological replicates. (D) Quantification of TfoS levels from western blots. For each replicate (n = 3), the normalized TfoS level was determined by dividing the intensity of the TfoS band by the intensity of the RpoA band. Results in A, B, and D are from three independent biological replicates and shown as the mean ± SD. Statistical comparisons in B and D were made by one-way ANOVA with Tukey’s multiple comparison test (normal distribution confirmed by Shapiro-Wilk test). Statistical identifiers directly above bars represent comparisons to the parent. ns, not significant. *** = p < 0.001, ** = p < 0.01. LOD, limit of detection. (TIFF) [file pgen.1011606.s002.tiff]

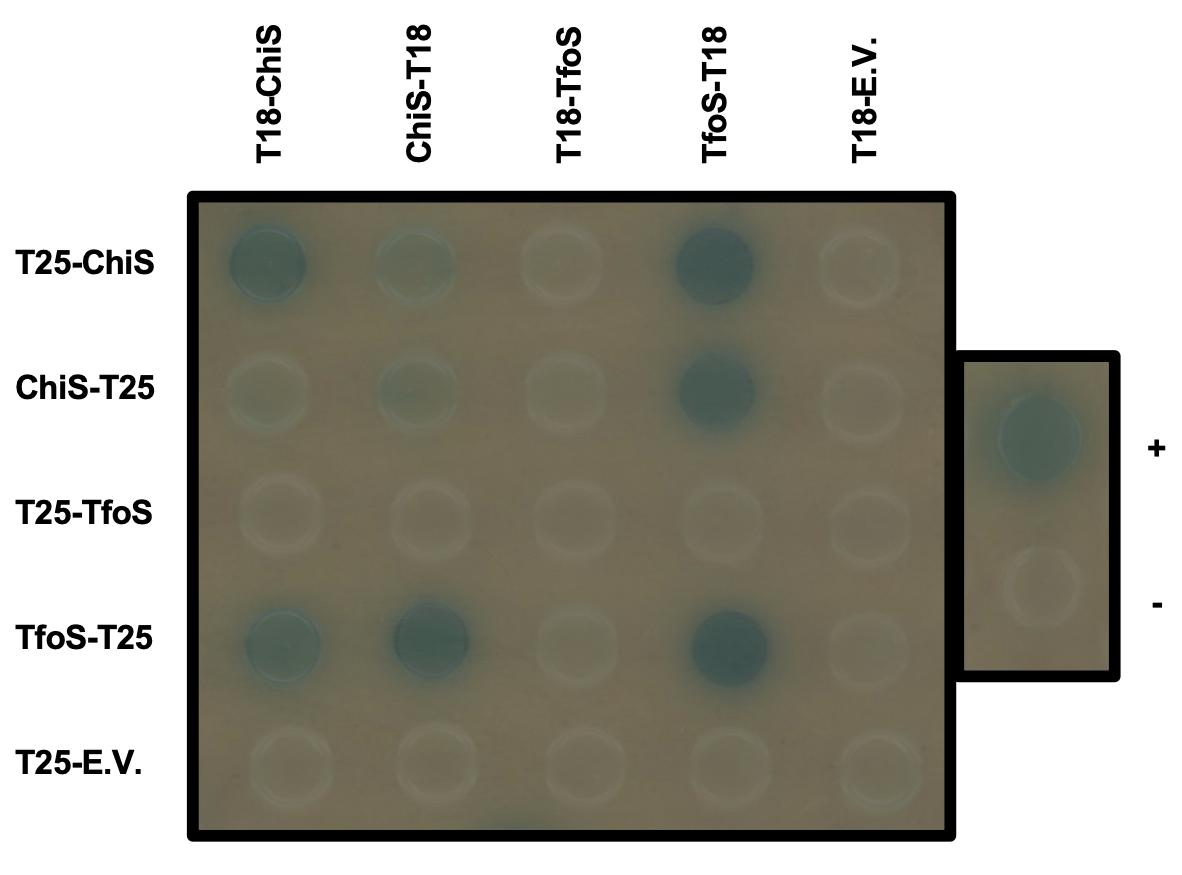

Supplement: S3 Fig — BACTH to assess interactions between ChiS and TfoS. For BACTH assays, ChiS and TfoS either had an N-terminal fusion (indicated as TXX-protein) or a C-terminal fusion (indicated as protein-TXX) to the T25 or T18 fragment of adenylate cyclase.“+” and “-” indicate positive (T18-zip + T25-zip) and negative (T18 E.V. + T25 E.V.) controls for the assay. “E.V.” denotes an empty vector. (TIFF) [file pgen.1011606.s003.tiff]

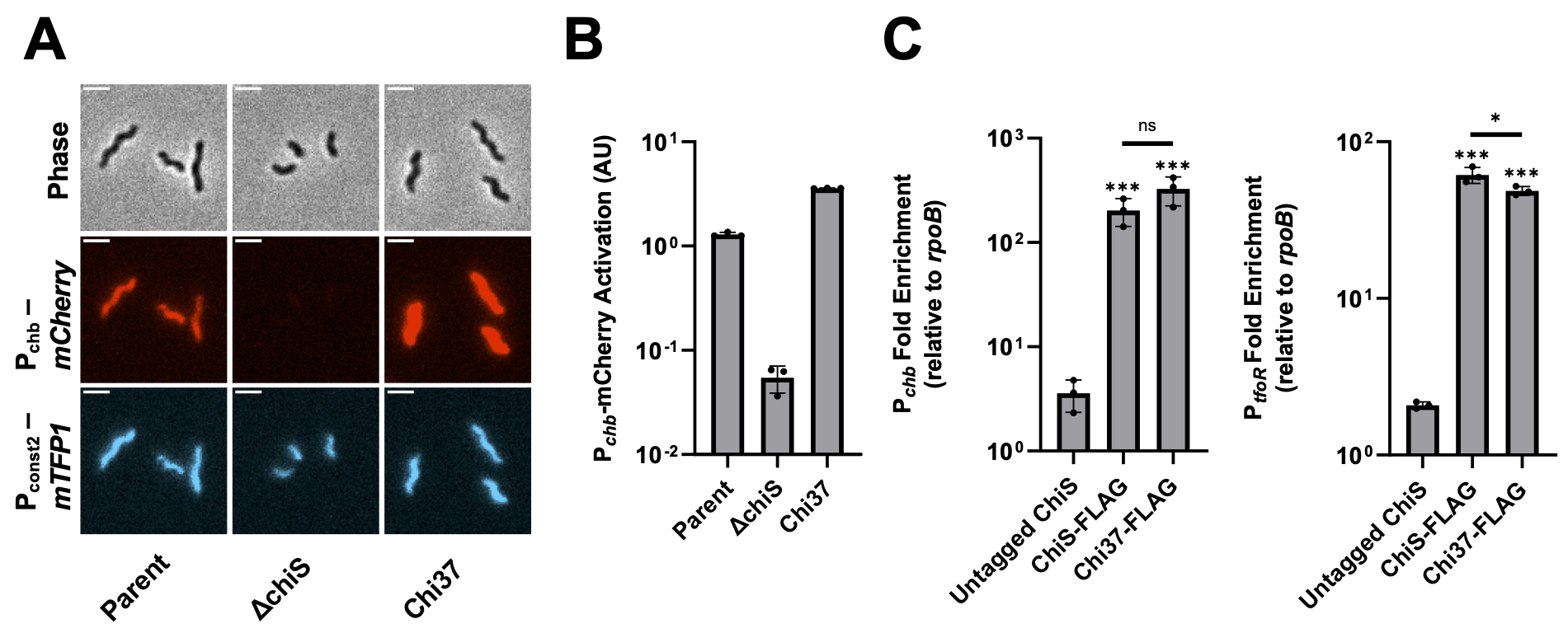

Supplement: S4 Fig — (A and B) Transcriptional reporter assays to assess chitin-dependent gene expression in the indicated strains. All strains harbored Pchb-mCherry and Pconst2-mTFP1 constructs. Cells were incubated on chitin and then imaged via epifluorescence microscopy to assess reporter expression. Representative images are shown in A and the quantification of the results are shown in B. For each replicate (n = 3), the geometric mean fluorescence was determined by analyzing 300 individual cells. (C) ChIP-qPCR assays were performed with the indicated strains to assess ChiS and Chi37 binding at Pchb and PtfoR in vivo. Results are from three independent biological replicates and shown as the mean ± SD. Statistical comparisons were made by one-way ANOVA with Tukey’s multiple comparison test (normal distribution confirmed by Shapiro-Wilk test). Statistical identifiers directly above bars represent comparisons to the parent. ns, not significant. *** = p < 0.001, * = p < 0.05. (TIFF) [file pgen.1011606.s004.tiff]

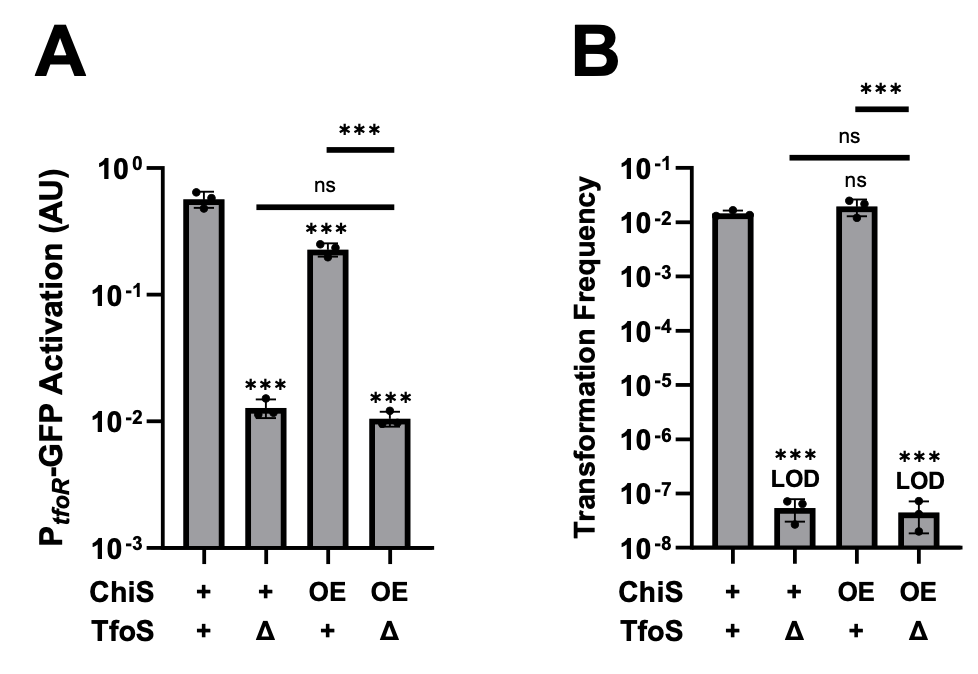

Supplement: S5 Fig — (A) Transcriptional reporter assays to assess chitin-dependent gene expression in the indicated strains. For ChiS genotypes, “+” denotes cells that have a WT copy of ChiS, and “OE” denotes that ChiS is overexpressed (Ptac-chiS + 1µM IPTG). For TfoS genotypes, “+” indicates that cells have a WT copy of TfoS, while “Δ” denotes that cells lack TfoS. All strains harbored PtfoR-gfp and Pconst2-mTFP1 constructs. For each replicate (n = 3), the geometric mean was determined by analyzing 300 individual cells. (B) Chitin-dependent transformation assays of the indicated strains. Results are from three independent biological replicates and shown as mean ± SD. Statistical comparisons were made by one-way ANOVA with Tukey’s multiple comparison test on the log-transformed data (normal distribution confirmed by Shapiro-Wilk test). Statistical identifiers directly above bars represent comparisons made to the parent. *** = p < 0.001, ns = not significant. LOD, limit of detection. (TIFF) [file pgen.1011606.s005.tiff]

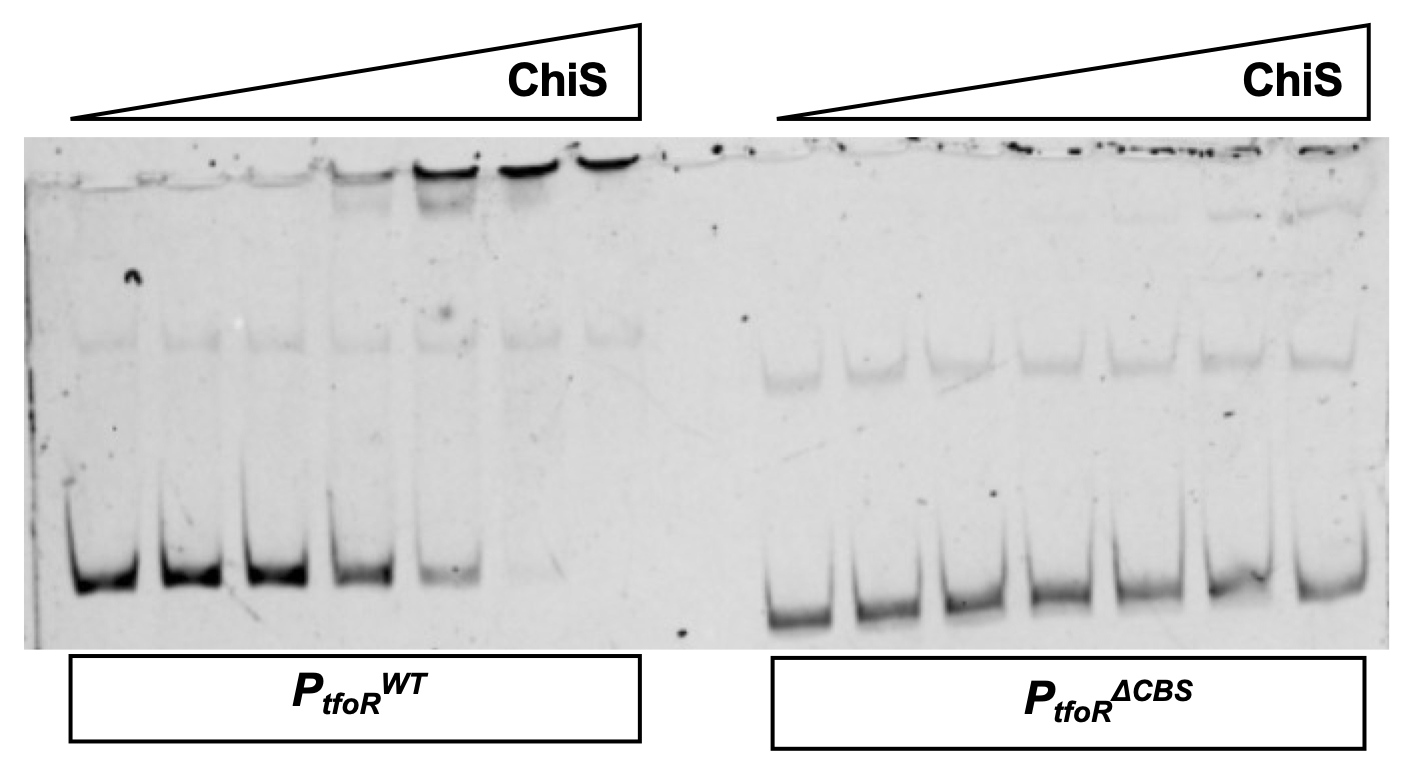

Supplement: S6 Fig — EMSAs of the purified ChiS cytoplasmic domain with the indicated probe DNA. Increasing concentrations of ChiS (from left to right: 0 nM, 25 nM, 50 nM, 100 nM, 200 nM, 400 nM, 800 nM) were incubated with the indicated Cy5-labelled PtfoR DNA probe. PtfoRWT denotes the full-length wildtype PtfoR promoter, while PtfoR∆CBS denotes the truncated promoter where the ChiS binding site was removed from PtfoR. Data are representative of three independent experiments. (TIFF) [file pgen.1011606.s006.tiff]

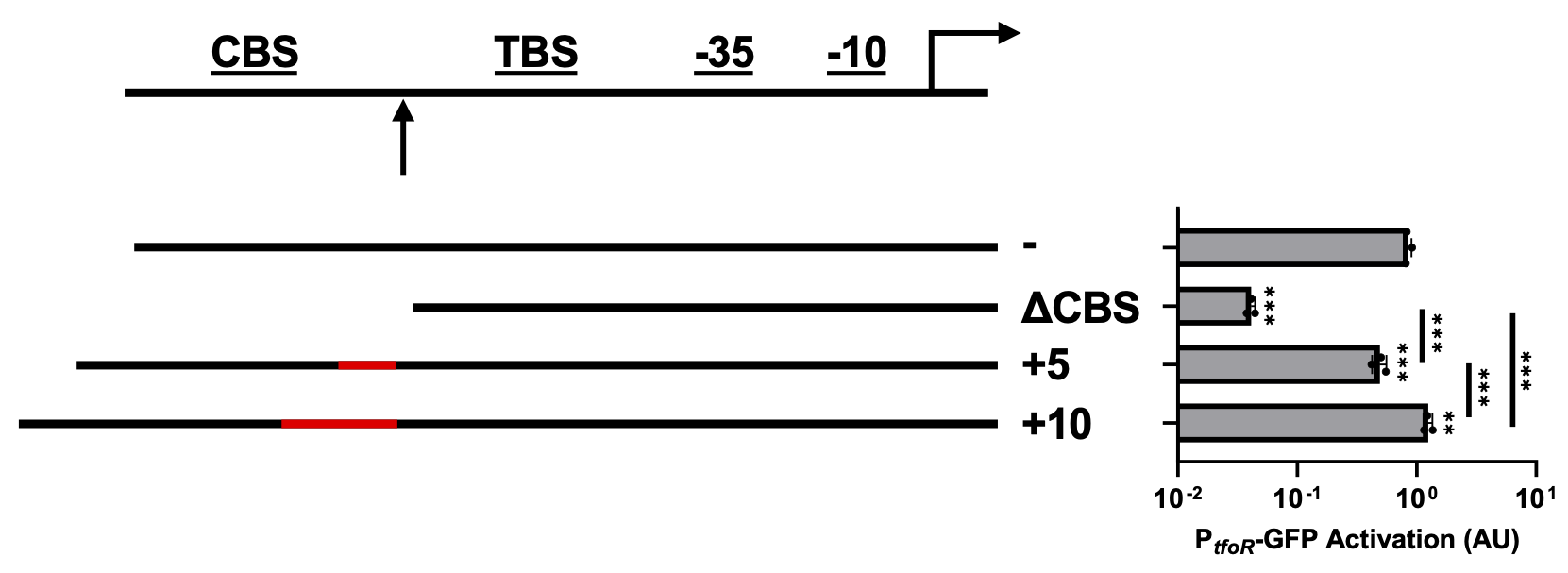

Supplement: S7 Fig — Transcriptional reporter assays to assess chitin-dependent gene expression of strains with the indicated PtfoR-gfp reporter constructs. Schematic of the PtfoR reporter constructs depicts where insertions of 5 bp or 10 bp (red) were introduced in between the ChiS binding site (CBS) and TfoS binding site (TBS) (see arrow) to alter the phasing between these elements. Activation of these reporters can be be compared to the parent PtfoR reporter (positive control) and the PtfoR∆CBS (negative control). Data are from three biological replicates (300 cells analyzed per replicate) and shown as the geometric mean ± SD. Statistical comparisons were made by one-way ANOVA with Tukey’s multiple comparison test on the log-transformed data (normal distribution confirmed by Shapiro-Wilk test). Statistical identifiers directly above bars represent comparisons made to the parent (top bar). ** = p < 0.01, *** = p < 0.001. (TIFF) [file pgen.1011606.s007.tiff]
