# Supplementary material for: Two transmembrane transcriptional regulators coordinate to activate chitin-induced natural transformation in Vibrio cholerae
Source: PLoS Genet. 2025 Feb 18;21(2):e1011606. doi: 10.1371/journal.pgen.1011606 (PMC11856585; doi:10.1371/journal.pgen.1011606)
Supplement: S1 Table — (PDF) [file pgen.1011606.s008.pdf]

Table S1. *Strains used in this study.*

Strain ID	Genotype	Reference in Manuscript
SAD030	*V. cholerae* WT E7946 Sm^R	Parent for all *V. cholerae* strains in this study<br><br>Fig. S1<br><br>ChiS+ TfoS+ TfoR+
SAD3634 / ACH0004	igVCA0265-66::Spec^R-P_{const2}-*mTFP1*; ΔVCA0692::Tm^R-P_{tfoR}-*gfp*; Δ*lacZ*::Kan^R-P_{chb}-*mCherry*	Fig. 1A-D<br><br>Parent
SAD3635 / ACH0014	igVCA0265-66::Spec^R-P_{const2}-*mTFP1*; ΔVCA0692::Tm^R-P_{tfoR}-*gfp*; Δ*lacZ*::Kan^R-P_{chb}-*mCherry*; Δ*tfoS*::Zeo^R	Fig. 1A-D<br><br>ΔtfoS
SAD3636 / ACH0019	igVCA0265-66::Spec^R-P_{const2}-*mTFP1*; ΔVCA0692::Tm^R-P_{tfoR}-*gfp*; Δ*lacZ*::Kan^R-P_{chb}-*mCherry*; Δ*chiS*::Carb^R	Fig. 1A-D, Fig. 3B-D, Fig. S4A-B<br><br>ΔchiS / ΔchiS TfoS+ P_{tfoR} CBS+
SAD3653 / ACH0205	Δ*crvA*::Zeo^R; igVCA0265-66::Cm^R-P_{bad}-*popZ*; Δ*lacZ*::Spec^R-P_{tac}-*tfoS*-L5-*mCherry*	Fig. 2<br><br>TfoS-mCherry only
SAD3652 / ACH0204	Δ*crvA*::Zeo^R; igVCA0265-66::Cm^R-P_{bad}-*popZ*; Δ*lacZ*::Spec^R-P_{tac}-*tfoS*-L5-*mCherry*; ΔVCA0692::Tm^R-P_{tac}-*chiS-msfGFP*-H3H4	Fig. 2<br><br>TfoS-mCherry ChiS-msfGFP-H3H4
SAD3654 / ACH0207	Δ*crvA*::Zeo^R; igVCA0265-66::Cm^R-P_{bad}-*popZ*; Δ*lacZ*::Spec^R-P_{tac}-*tfoS*-L5-*mCherry*; ΔVCA0692::Tm^R-P_{tac}-*chi37-msfGFP*-H3H4	Fig. 2<br><br>TfoS-mCherry Chi37-msfGFP-H3H4
SAD3645 / ACH0142	igVCA0265-66::Spec^R-P_{const2}-*mTFP1*; ΔVCA0692::Tm^R-P_{tfoR}-*gfp*; Δ*lacZ*::Kan^R-P_{chb}-*mCherry*; Δ*chiS*::Carb^R; igVCA0587-88::Erm^R-P_{chiS}-*chiS*	Fig. 3B-D ; Fig. 4B-D ; Fig. S4A-B ; Fig. S5<br><br>ChiS+ TfoS+ P_{tfoR} CBS+
SAD3655 / ACH0273	igVCA0265-66::Spec^R-P_{const2}-*mTFP1*; ΔVCA0692::Tm^R-P_{tfoR}-*gfp*; Δ*lacZ*::Kan^R-P_{chb}-*mCherry*; Δ*chiS*::Carb^R; igVCA0587-88::Erm^R-P_{chiS}-*chiS*; Δ*tfoS*::Zeo^R	Fig. 3B-D ; Fig. S5<br><br>ChiS+ ΔtfoS P_{tfoR} CBS+
SAD3644 / ACH0141	igVCA0265-66::Spec^R-P_{const2}-*mTFP1*; ΔVCA0692::Tm^R-P_{tfoR}-*gfp*; Δ*lacZ*::Kan^R-P_{chb}-*mCherry*; Δ*chiS*::Carb^R; igVCA0587-88::Erm^R-P_{chiS}-*chi37*	Fig. 3B-D ; Fig. S4A-B<br><br>Chi37 TfoS+ P_{tfoR} CBS+
SAD3647 / ACH0144	igVCA0265-66::Spec^R-P_{const2}-*mTFP1*; ΔVCA0692::Tm^R-P_{tfoR}-*gfp*; Δ*chiS*::Carb^R; igVCA0587-88::Erm^R-P_{chiS}-*chiS*; Δ*lacZ*::Zeo^R-P_{tac}-*tfoS*	Fig. 3B-D<br><br>ChiS+ TfoS OE P_{tfoR} CBS+
SAD3646 / ACH0143	igVCA0265-66::Spec^R-P_{const2}-*mTFP1*; ΔVCA0692::Tm^R-P_{tfoR}-*gfp*; Δ*chiS*::Carb^R; igVCA0587-88::Erm^R-P_{chiS}-*chi37*; Δ*lacZ*::Zeo^R-P_{tac}-*tfoS*	Fig. 3B-D<br><br>Chi37 TfoS OE P_{tfoR} CBS+
SAD3648 / ACH0147	igVCA0265-66::Spec^R-P_{const2}-*mTFP1*; ΔVCA0692::Tm^R-P_{tfoR}-*gfp*; Δ*chiS*::Carb^R; Δ*lacZ*::Zeo^R-P_{tac}-*tfoS*	Fig. 3B-D<br><br>ΔchiS TfoS OE P_{tfoR} CBS+
SAD3678 / ACH0499	igVCA0265-66::Spec^R-P_{const2}-*mTFP1*; ΔVCA0692::Tm^R-$P_{tfoR}^{\Delta CBS}$-*gfp*; $P_{tfoR}^{\Delta CBS}$; Δ*chiS*::Carb^R; igVCA0587-88::Erm^R-P_{chiS}-*chiS*	Fig. 3B-C<br><br>ChiS+ TfoS+ ΔP_{tfoR} CBS

SAD3679 / ACH0500	igVCA0265-66::Spec^{R}-P$_{const2}$-*mTFP1*; ΔVCA0692::Tm^{R}-P$_{tfoR}^{\Delta CBS}$-*gfp*; P$_{tfoR}^{\Delta CBS}$; Δ*chiS*::Carb^{R}; igVCA0587-88::Erm^{R}-P$_{chiS}$-*chiS*; Δ*lacZ*::Zeo^{R}-P$_{tac}$-*tfoS*	Fig. 3B-C<br><br>ChiS+ TfoS OE ΔP$_{tfoR}$ CBS
SAD3681 / ACH0504	igVCA0265-66::Spec^{R}-P$_{const2}$-*mTFP1*; ΔVCA0692::Tm^{R}-P$_{tfoR}^{\Delta CBS}$-*gfp*; P$_{tfoR}^{\Delta CBS}$; Δ*chiS*::Carb^{R}; igVCA0587-88::Erm^{R}-P$_{chiS}$-*chi37*	Fig. 3B-C<br><br>Chi37 TfoS+ ΔP$_{tfoR}$ CBS
SAD3680 / ACH0502	igVCA0265-66::Spec^{R}-P$_{const2}$-*mTFP1*; ΔVCA0692::Tm^{R}-P$_{tfoR}^{\Delta CBS}$-*gfp*; P$_{tfoR}^{\Delta CBS}$; Δ*chiS*::Carb^{R}; igVCA0587-88::Erm^{R}-P$_{chiS}$-*chi37*; Δ*lacZ*::Zeo^{R}-P$_{tac}$-*tfoS*	Fig. 3B-C<br><br>Chi37 TfoS OE ΔP$_{tfoR}$ CBS
SAD3656 / ACH0278	igVCA0265-66::Spec^{R}-P$_{const2}$-*mTFP1*; ΔVCA0692::Tm^{R}-P$_{tfoR}$-*gfp*; Δ*lacZ*::Kan^{R}-P$_{chb}$-*mCherry*; Δ*chiS*::Carb^{R}; igVCA0587-88::Erm^{R}-P$_{chiS}$-*chi37*; P$_{tfoR}^{\Delta CBS}$; ΔVC1807::Smx^{R}	Fig. 3D<br><br>Chi37 TfoS+ ΔP$_{tfoR}$ CBS
SAD3657 / ACH0279	igVCA0265-66::SpecR-P$_{const2}$-*mTFP1*; ΔVCA0692::TmR-P$_{tfoR}$-*gfp*; ΔlacZ::KanR-P$_{chb}$-*mCherry*; Δ*chiS*::CarbR; igVCA0587-88::ErmR-P$_{chiS}$-*chiS*; P$_{tfoR}^{\Delta CBS}$; ΔVC1807::SmxR	Fig. 3D<br><br>ChiS+ TfoS+ ΔP$_{tfoR}$ CBS
SAD3658 / ACH0280	igVCA0265-66::Spec^{R}-P$_{const2}$-*mTFP1*; ΔVCA0692::Tm^{R}-P$_{tfoR}$-*gfp*; Δ*chiS*::Carb^{R}; igVCA0587-88::Erm^{R}-P$_{chiS}$-*chi37*; P$_{tfoR}^{\Delta CBS}$; ΔVC1807::Smx^{R}; Δ*lacZ*::Zeo^{R}-P$_{tac}$-*tfoS*	Fig. 3D<br><br>Chi37 TfoS OE ΔP$_{tfoR}$ CBS
SAD3659 / ACH0281	igVCA0265-66::Spec^{R}-P$_{const2}$-*mTFP1*; ΔVCA0692::Tm^{R}-P$_{tfoR}$-*gfp*; Δ*chiS*::Carb^{R}; igVCA0587-88::Erm^{R}-P$_{chiS}$-*chiS*; P$_{tfoR}^{\Delta CBS}$; ΔVC1807::Smx^{R}; Δ*lacZ*::Zeo^{R}-P$_{tac}$-*tfoS*	Fig. 3D<br><br>ChiS+ TfoS OE ΔP$_{tfoR}$ CBS
SAD3670 / ACH0374	igVCA0265-66::Spec^{R}-P$_{const2}$-*mTFP1*; ΔVCA0692::Tm^{R}-P$_{tfoR}^{\Delta CBS::tetO}$-*gfp*; Δ*lacZ*::Kan^{R}-P$_{chb}$-*mCherry*; Δ*chiS*::Carb^{R}; igVCA0587-88::Erm^{R}-P$_{chiS}$-*chiS*	Fig. 4B-C<br><br>ChiS+ TfoS+ P$_{tfoR}$ CBS tetO
SAD3666 / ACH0310	igVCA0265-66::Spec^{R}-P$_{const2}$-*mTFP1*; ΔVCA0692::Tm^{R}-P$_{tfoR}^{\Delta CBS::tetO}$-*gfp*; Δ*lacZ*::Kan^{R}-P$_{chb}$-*mCherry*; Δ*chiS*::Carb^{R}; igVCA0587-88::Erm^{R}-P$_{chiS}$-*chiS*$^{\Delta DBD}$-*TetR*	Fig. 4B-C<br><br>ChiS TetR TfoS+ P$_{tfoR}$ CBS tetO
SAD3667 / ACH0325	igVCA0265-66::Spec^{R}-P$_{const2}$-*mTFP1*; ΔVCA0692::Tm^{R}-P$_{tfoR}^{\Delta CBS::tetO}$-*gfp*; Δ*lacZ*::Kan^{R}-P$_{chb}$-*mCherry*; Δ*chiS*::Carb^{R}	Fig. 4B-C<br><br>ΔchiS TfoS+ P$_{tfoR}$ CBS tetO
SAD3668 / ACH0338	igVCA0265-66::Spec^{R}-P$_{const2}$-*mTFP1*; ΔVCA0692::Tm^{R}-P$_{tfoR}^{\Delta CBS::tetO}$-*gfp*; Δ*chiS*::Carb^{R}; igVCA0587-88::Erm^{R}-P$_{chiS}$-*chiS*$^{\Delta DBD}$-*TetR*; Δ*lacZ*::Zeo^{R}-P$_{tac}$-*tfoS*	Fig. 4B-C<br><br>ChiS TetR TfoS OE P$_{tfoR}$ CBS tetO
SAD3669 / ACH0340	igVCA0265-66::Spec^{R}-P$_{const2}$-*mTFP1*; ΔVCA0692::Tm^{R}-P$_{tfoR}^{\Delta CBS::tetO}$-*gfp*; Δ*lacZ*::Kan^{R}-P$_{chb}$-*mCherry*; Δ*chiS*::Carb^{R}; Δ*lacZ*::Zeo^{R}-P$_{tac}$-*tfoS*	Fig. 4B-C<br><br>ΔchiS TfoS OE P$_{tfoR}$ CBS tetO
SAD3674 / ACH0401	Δ*chiS*::Carb^{R}; igVCA0587-88::Erm^{R}-P$_{chiS}$-*chiS*; P$_{tfoR}^{\Delta CBS::tetO}$; ΔVC1807::Smx^{R}	Fig. 4D<br><br>ChiS+ TfoS+ P$_{tfoR}$ CBS tetO

SAD3672 / ACH0399	Δ*chiS*::Carb^{R}; igVCA0587-88::Erm^{R}-P$_{chiS}$-*chiS*$^{\Delta DBD}$-*TetR*; P$_{tfoR}^{\Delta CBS::tetO}$; ΔVC1807::Smx^{R}	Fig. 4D<br><br>ChiS TetR TfoS+ P$_{tfoR}$ CBS tetO
SAD3671 / ACH0398	Δ*chiS*::Carb^{R}; P$_{tfoR}^{\Delta CBS::tetO}$; ΔVC1807::Smx^{R}	Fig. 4D<br><br>ΔchiS TfoS+ P$_{tfoR}$ CBS tetO
SAD3673 / ACH0400	Δ*chiS*::Carb^{R}; igVCA0587-88::Erm^{R}-P$_{chiS}$-*chiS*$^{\Delta DBD}$-*TetR*; P$_{tfoR}^{\Delta CBS::tetO}$; ΔVC1807::Smx^{R}; Δ*lacZ*::Zeo^{R}-P$_{tac}$-*tfoS*	Fig. 4D<br><br>ChiS TetR TfoS OE P$_{tfoR}$ CBS tetO
SAD3677 / ACH0418	Δ*chiS*::Carb^{R}; P$_{tfoR}^{\Delta CBS::tetO}$; ΔVC1807::Smx^{R}; Δ*lacZ*::Zeo^{R}-P$_{tac}$-*tfoS*	Fig. 4D<br><br>ΔchiS TfoS OE P$_{tfoR}$ CBS tetO
SAD3662 / ACH0288	Δ*chiS*::Carb^{R}	Fig. S1<br><br>ΔchiS TfoS+ TfoR+
SAD3663 / ACH0289	Δ*tfoS*::Zeo^{R}	Fig. S1<br><br>ChiS+ ΔtfoS TfoR+
SAD3664 / ACH0290	Δ*chiS*::Carb^{R}; Δ*lacZ*::Spec^{R}-P$_{tac}$-*tfoR*	Fig. S1<br><br>ΔchiS TfoS+ TfoR OE
SAD3665 / ACH0291	Δ*tfoS*::Zeo^{R}; Δ*lacZ*::Spec^{R}-P$_{tac}$-*tfoR*	Fig. S1<br><br>ChiS+ ΔtfoS TfoR OE
SAD3650 / ACH0186	igVCA0265-66::Spec^{R}-P$_{const2}$-*mTFP1*; Δ*lacZ*::Kan^{R}-P$_{tfoR}$-*gfp*; ΔVC1807::Cm^{R}	Fig. S2<br><br>Untagged
SAD3649 / ACH0185	igVCA0265-66::Spec^{R}-P$_{const2}$-*mTFP1*; Δ*lacZ*::Kan^{R}-P$_{tfoR}$-*gfp*; ΔVC1807::Cm^{R}; *tfoS* N929 1X FLAG	Fig. S2<br><br>TfoS-FLAG
SAD3651 / ACH0189	igVCA0265-66::Spec^{R}-P$_{const2}$-*mTFP1*; Δ*lacZ*::Kan^{R}-P$_{tfoR}$-*gfp*; ΔVC1807::Cm^{R}; *tfoS* N929 1X FLAG; Δ*chiS*::Carb^{R}	Fig. S2<br><br>TfoS-FLAG ΔchiS
SAD3661 / ACH0286	igVCA0265-66::Spec^{R}-P$_{const2}$-*mTFP1*; ΔVC1807::Cm^{R}; Δ*lacZ*::Zeo^{R}-P$_{tac}$-*tfoS N929 1X FLAG*	Fig. S2<br><br>Ptac-TfoS-FLAG
SAD3637 / ACH0031	TG1, pKT25-ChiS Kan^{R}	Fig. S3<br><br>T25-ChiS
SAD3640 / ACH0035	TG1, pKT25-TfoS Kan^{R}	Fig. S3<br><br>T25-TfoS
SAD3683 / VEG0253	TG1, pKNT25-ChiS Kan^{R}	Fig. S3<br><br>ChiS-T25
SAD3641 / ACH0036	TG1, pKNT25-TfoS Kan^{R}	Fig. S3<br><br>TfoS-T25
SAD3638 / ACH0033	TG1, pUT18C-ChiS Carb^{R}	Fig. S3<br><br>T18-ChiS
SAD3642 / ACH0037	TG1, pUT18C-TfoS Carb^{R}	Fig. S3<br><br>T18-TfoS

SAD3639 / ACH0034	TG1, pUT18-ChiS Carb^R	Fig. S3<br><br>ChiS-T18
SAD3643 / ACH0038	TG1, pUT18-TfoS Carb^R	Fig. S3<br><br>TfoS-T18
SAD2236	TG1, pKT25 vector Kan^R	Fig. S3<br><br>T25-E.V.
SAD2240	TG1, pUT18C vector Carb^R	Fig. S3<br><br>T18-E.V.
SAD2238	TG1, pKT25-zip Kan^R	Fig. S3<br><br>T25-zip
SAD2241	TG1, pUT18C-*zip* Carb^R	Fig. S3<br><br>T18-zip
SAD3354 / VEG0565	Δ*lacZ*::Kan^R-P$_{chb}$-*gfp*; Δ*chiS/cbp*::Carb^R; ΔVCA0692::*Tm*^{R}-P$_{chiS}$-*chiS*	Fig. S4C<br><br>Untagged ChiS
SAD2706 / TND1801	Δ*lacZ*::Kan^R-P$_{chb}$-*gfp*; Δ*chiS/cbp*::Carb^R; ΔVCA0692::Tm^R-P$_{chiS}$-*chiS* 1X FLAG after E566	Fig. S4C<br><br>ChiS-FLAG
SAD3682 / CAK672	Δ*lacZ*::Kan^R-P$_{chb}$-*gfp*; Δ*chiS/cbp*::Carb^R; ΔVCA0692::Tm^R-P$_{chiS}$-*chi37* 3X FLAG	Fig. S4C<br><br>Chi37-FLAG
SAD3675 / ACH0411	igVCA0265-66::Spec^R-P$_{const2}$-*mTFP1*; ΔVCA0692::Tm^R-P$_{tfoR}$-*gfp*; Δ*lacZ*::Kan^R-P$_{chb}$-*mCherry*; Δ*chiS*::Carb^R; igVCA0587-88::Erm^R-P$_{tac}$-*chiS E566 1X FLAG*	Fig. S5<br><br>ChiS OE TfoS+
SAD3676 / ACH0414	igVCA0265-66::Spec^R-P$_{const2}$-*mTFP1*; ΔVCA0692::Tm^R-P$_{tfoR}$-*gfp*; Δ*lacZ*::Kan^R-P$_{chb}$-*mCherry*; Δ*chiS*::Carb^R; igVCA0587-88::Erm^R-P$_{tac}$-*chiS E566 1X FLAG*; Δ*tfoS*::Zeo^R	Fig. S5<br><br>ChiS OE ΔtfoS
SAD3684 / TND4270	igVCA0265-66::Spec^R-P$_{const2}$-*mTFP1*; ΔVCA0692::Tm^R-P$_{tfoR}$-*gfp*	Fig. S7<br><br>-
SAD3685 / TND4271	igVCA0265-66::Spec^R-P$_{const2}$-*mTFP1*; ΔVCA0692::Tm^R-P$_{tfoR}^{+5bp}$-*gfp*	Fig. S7<br><br>+5
SAD3686 / TND4272	igVCA0265-66::Spec^R-P$_{const2}$-*mTFP1*; ΔVCA0692::Tm^R-P$_{tfoR}^{+10bp}$-*gfp*	Fig. S7<br><br>+10
SAD3660 / ACH0284	igVCA0265-66::Spec^R-P$_{const2}$-*mTFP1*; ΔVCA0692::Tm^R-P$_{tfoR}^{\Delta CBS}$-*gfp*; Δ*chiS*::Carb^R; igVCA0587-88::Erm^R-P$_{chiS}$-*chiS*	Fig. S7<br><br>ΔCBS

Double identifiers under "Strain ID" refer to the same strain that has been stocked in two independent strain collections.
