# Supplementary material for: Two transmembrane transcriptional regulators coordinate to activate chitin-induced natural transformation in Vibrio cholerae
Source: PLoS Genet. 2025 Feb 18;21(2):e1011606. doi: 10.1371/journal.pgen.1011606 (PMC11856585; doi:10.1371/journal.pgen.1011606)
Supplement: S2 Table — (PDF) [file pgen.1011606.s009.pdf]

Table S2. *Primers used in this study.*

Primers for SOE Deletions		
**Primer**	**Sequence**	**Description**
ABD123	ATTCCGGGGATCCGTCGAC	Antibiotic resistance cassette F
ABD124	TGTAGGCTGGAGCTGCTTC	Antibiotic resistance cassette R
ABD767	TTAATTTGGATCCCTGCGACACTC	ΔchiS F1 for Up arm
ABD768	gtcgacggatccccggaatCAAAAAACGTGAGGAGA ATGCC	ΔchiS R1 for Up arm
ABD769	gaagcagctccagcctacaTTCTTGAGCATTGCAAAG AAGC	ΔchiS F2 for Down arm
ABD770	CTGGAACGAATGAAGAAGTCCAG	ΔchiS R2 for Down arm
ABD640	GCAATACACGCTGTGTTTCACCG	ΔtfoS F1 for Up arm
ABD641	gtcgacggatccccggaatCAATTCAAGAATTCGTGCTTTATTGG	ΔtfoS R1 for Up arm
ABD642	gaagcagctccagcctacaTAATAGAGCCCATCTCTATTTCATC	ΔtfoS F2 for Down arm
ABD643	TCGTTTTCTAAGGCTCGAATCGCC	ΔtfoS R2 for Down arm
BBC1782	CAAGCGATGTACGAACGAAC	ΔcrvA F1 for Up arm
BBC1783	gtcgacggatccccggaatCCACATAAAGTGGGAAAGACAAAC	ΔcrvA R1 for Up arm
BBC1784	gaagcagctccagcctacaGTCAATGGCAATGACACGG	ΔcrvA F2 for Down arm
BBC1785	GCTTGACGTTGTTGCTTACTGG	ΔcrvA R2 for Down arm
BBC717	AAATAGATTTGGTGACTTTACCTCC	ΔVC1807 F1 for UP arm
ABD340	gtcgacggatccccggaatACGTTTCATTAGTCACCTCTATTGTTAA CTTGTTC	ΔVC1807 R1 for Up arm
ABD341	gaagcagctccagcctacaTAGTCGAAAATAAAAAAAAGAGGCTC GCCTC	ΔVC1807 F2 for Down arm
BBC2412	CAATTTTGCTTTTGGACCATCC	ΔVC1807 R2 for Down arm
ABD725	GAAGCAGCTCCAGCCTACA	Detect F for all deletions
CKP001	cccgggatcctgtgtgaaattgCTTATTTGGCCTTGTTTGATAATG	ΔchiS detect R
CKP682	AATAGTCAACGTCAATTCTGTC	ΔtfoS detect R
BBC1786	TTTTGTGATGGCTGGATGTCG	ΔcrvA detect R
BBC030	ACCAAACAATAAACGAGTAATGC	ΔVC1807 detect R
**Primers for Reporter Constructs**		
**Primer**	**Sequence**	**Description**
BBC3230	GCAGTAAATCCGACTTTGGAG	Insert construct at igVCA0265-66 F1
BBC3260	CCCGGGATCCTGTGTGAAATTG	igVCA0265-66 $Spec^R$ R1 for P_{const2}
BBC4271	caatttcacacaggatcccgggAGGAGGTtGTGTTATGGTGAGCAAA GGTG	Amplify mTFP1 F
BBC4272	tgtaggctggagctgcttcCTATTTATACAGTTCATCCATACCATC	Amplify mTFP1 R
CKP803	gaagcagctccagcctacaTCATAATTTAAGGCGTTAGCAG	Insert construct at igVCA0265-66 F2
BBC3231	TCTCATAAACCTCATGGTAGACG	Insert construct at igVCA0265-66 R2

BBC832	GCTTTTTGCTACAACGACCG	Insert construct at VCA0692 F1
BBC263	TACCGAGGACGCGAAGCTGCTCATTAGGCACCCCAGGC	Insert construct at VCA0692 R1
CKP699	cagcttcgcgtcctcggtaAATAGTCAACGTCAATTCTGTC	Amplify PtfoR-GFP F
BBC254	tgtaggctggagctgcttcTTAGTTGTATAGTTCATCCATGCC	Amplify PtfoR-GFP R
BBC830	gaagcagctccagcctacaGTTGAGTTGGATGCAGCACC	Insert construct at VCA0692 F2
BBC834	CACAATTTCTCGCTTAAAATGTCC	Insert construct at VCA0692 R2
BBC2511	CTCATTAGGCACCCCAGGC	PtfoR$^{\Delta CBS}$-GFP R1
BBC4082	gcctggggtgcctaatgagTAAAATCAACACCTTAAAAACATGATTAAGC	PtfoR$^{\Delta CBS}$-GFP F2
VGP0466	tctctatcactgatagggaTAgAATAGGTACTAATCAgAATAGTGAGGAG	PtfoR$^{\Delta CBS::tetO}$-GFP R1
VGP0467	tccctatcagtgatagagaTCACATTACCATTAAgATGTCAAACGG	PtfoR$^{\Delta CBS::tetO}$-GFP F2
VGP0468	ctcctcactattctgattagtacctattctaTCCCTATCAGTGATAGAGAttcttTCCCTATCAGTGATAGAGAttcttTCCCTATCAGTGATAGAGAatcgtTCCCTATCAGTGATAGAGAtcacattaccattaagatgtcaaacgg	4X tetO miniFRT F
VGP0469	ccgtttgacatcttaatggtaatgtgaTCTCTATCACTGATAGGGAacgatTCTCTATCACTGATAGGGAaagaaTCTCTATCACTGATAGGGAaagaaTCTCTATCACTGATAGGGAtagaataggtactaatcagaatagtgaggag	4X tetO miniFRT R
BBC4945	TTTTCTGACTTGGTCTTTGACG	Insert X bp between CBS and TBS in PtoR-GFP R1
BBC4946	CGTCAAAGACCAAGTCAGAAAAtttttGTCTCCTGCATAAAATCAACATG	PtfoR^{+5}-GFP F2
BBC4947	CGTCAAAGACCAAGTCAGAAAAttttttttttGTCTCCTGCATAAAATCAACATG	PtfoR^{+10}-GFP F2
**Primers for BACTH**		
**Primer**	**Sequence**	**Description**
BBC2513	gaagcagctccagcctacaGATCCCCGGGTACCTAAGTAAC	Amplify BACTH pKT25 to make T25-XX vectors F
BBC2512	tccacctgctccacctgcCTCTAGAGTCGACCCTGCAG	Amplify BACTH pKT25 to make T25-XX vectors R
BBC2515	gaagcagctccagcctacaCCGAGCTCGAATTCATCGATATAAC	Amplify BACTH pUT18C to make T18-XX vectors F
BBC2514	tccacctgctccacctgcCTCTAGAGTCGACCTGCAG	Amplify BACTH pUT18C to make T18-XX vectors R
BBC2517	gcaggtggaagtggtggaGATCCCCGGGTACCGAGC	Amplify BACTH pKNT25 and pUT18 to make XX-T25 and XX-T18 vectors F
BBC2516	tccacctgctccacctgcCTCTAGAGTCGACCTGCAGG	Amplify BACTH pKNT25 and pUT18 to make XX-T25 and XX-T18 vectors R

BBC2762	gcaggtggagcaggtggaTTTAGGTTCTATCGAAAACAAAAATTC	BACTH TXX-ChiS or ChiS-TXX F
BBC3203	tgtaggctggagctgcttcTTATTCACTGGTCAGGAGTTTTTGC	BACTH TXX-ChiS R
BBC3204	tccaccacttccacctgcTTCACTGGTCAGGAGTTTTTGC	BACTH ChiS-TXX R
VGP0213	gcaggtggagcaggtggaTCGCTGACAGGGGAACTCG	BACTH TXX-TfoS or TfoS-TXX F
VGP0214	tgtaggctggagctgcttcTTAGTTTTCTTGATCTTCGATGAATTG	BACTH TXX-TfoS R
VEG0215	tccaccacttccacctgcGTTTTCTTGATCTTCGATGAATTGG	BACTH TfoS-TXX R
**Primers for EMSAs**		
**Primer**	**Sequence**	**Description**
BBC4084	gtcgacggatccccggaatCCGGTGATATTCTCATTTTAGC	P_{tfoR}^{WT} F from Fig. 1F
CKP235	gtcgacggatccccggaatCAAATATATCCTCCTCACTATTTTGATTAG	P_{tfoR}^{WT} and $P_{tfoR}^{\Delta CBS}$ F from Fig. S5
BBC929	AACATCACCATCTAATTCAACAAG	P_{tfoR}^{WT} and $P_{tfoR}^{\Delta CBS}$ R
CKP248	TGTACTGTACAAATATTTTTTTTGTACAG	$P_{VCA0053}$ F
CKP249	TGTGGGGTTGCCATGGTTAC	$P_{VCA0053}$ R
**Primers for ChiS Constructs**		
**Primer**	**Sequence**	**Description**
BBC4387	GATAACGATCGATAGCTGGTTGG	Insert construct at igVCA0587-88 F1
BBC647	tttttctatttctgaatcgattcatacgaCTCATTAGGCACCCCAGGC	igVCA0587-88 Erm^{R} R1
BBC1889	tcgtatgaatcgattcagaaatagaaaaaTTTGCCGCTTTTAACGTAAATCAG	Amplify PchiS-ChiS F
BBC577	tgtaggctggagctgcttcTTATTCACTGGTCAGGAGTTTTTGC	Amplify PchiS-ChiS R
BBC4384	gaagcagctccagcctacaGCAGTGTTGGCTGCAACTCC	Insert construct at igVCA0587-88 F2
BBC4388	ATCATGGTGCTGTTTAGTTGG	Insert construct at igVCA0587-88 R2
CAK311	ATTTTATGGCTGAATTTCATAATTAAGGTGTTACTTTCGGTG	Mlp37 periplasmic domain stitch to PchiS R1
CAK312	CCGAAAGTAACACCTTAATTATGAAATTCAGCCATAAAATTGTTG	Mlp37 periplasmic domain stitch to PchiS F
CKP384	GCGGTGAGGTAATGAAATTAACGATAAACAGTAGAACAAACACG	Mlp37 periplasmic domain R
CKP385	TTCTACTGTTTATCGTTAATTTCATTACCTCACCGCTTTTG	ChiS HAMP + cytoplasmic stitch to Mlp37 periplasmic F2
CKP397	ataatcaatgtcgtgatccttgtagtcaccatcatggtctttataatcATCGACAATCTCATCAACACTG	Mlp37 periplasmic domain truncation 3X FLAG R1
CKP396	ggtgactacaaggatcacgacattgattataaggatgacgatgacaaaGAATTTTTGGGTGAAAGCAAAATC	Mlp37 periplasmic domain truncation 3X FLAG F2
BBC4756	gccagctttcgcggcagcttcgccaccagaaccgctTTGATACTCGCCCGAGGCTT	Amplify $ChiS^{\Delta DBD}$ R1
BBC4757	agcggttctggtggcgaagctgccgcgaaagctggcGTGTCTAGATTAGATAAAAGTAAAGTG	Amplify TetR F
VGP0470	tgtaggctggagctgcttcTTAtccacctgctccacctgc	Amplify TetR R

Primers for POLAR Constructs		
**Primer**	**Sequence**	**Description**
ABD332	GGCTGAACGTGGTTGTCGAAAATGAC	Insert construct at *lacZ* F1
BBC3668	cagctttcgcggcagcttcgccaccagaaccgctGTTTTCTTGATCTTCGATGAATTGGC	Amplify TfoS R1
BBC3669	ttctggtggcgaagctgccgcgaaagctggcGTGagcaagggcgaggaggataacatgg	Amplify mCherry F
BBC206	tgtaggctggagctgcttcttacttgtacagctcgtccatg	Amplify mCherry R
ABD255	gaagcagctccagcctacaCCACAATAAGCCAGAGAGCCTTAAG	Insert construct at *lacZ* F2
ABD256	CCCAAATACGGCAACTTGGCG	Insert construct at *lacZ* R2
**Primers for TfoS Constructs**		
**Primer**	**Sequence**	**Description**
ABD332	GGCTGAACGTGGTTGTCGAAAATGAC	Insert construct at *lacZ* F1
BBC244	CCCGGGATCCTGTGTGAAATTGTTATCCGC	Insert construct at *lacZ* Zeo^R R1
BBC4343	caatttcacacaggatcccgggAGGAGGTGAACTCaTGTTTCGCACATTG	Amplify TfoS for Ptac F
BBC2476	tgtaggctggagctgcttcTTAGTTTTCTTGATCTTCGATGAATTGGC	Amplify TfoS for Ptac R
ABD255	gaagcagctccagcctacaCCACAATAAGCCAGAGAGCCTTAAG	Insert construct at *lacZ* F2
ABD256	CCCAAATACGGCAACTTGGCG	Insert construct at *lacZ* R2
ABD640	GCAATACACGCTGTGTTTCACCG	Amplify native TfoS F1
CKP760	tccaccacttccacctgcATTTCTCACTTCAAGTTGTATGG	Insert tag after TfoS N929 R1
BBC2274	gcaggtggaagtggtggagattataaggatgacgatgacaaagcaggtggagcaggtgga	1X FLAG F
BBC2275	tccacctgctccacctgctttgtcatcgtcatccttataatctccaccacttccacctgc	1X FLAG R
CKP761	gcaggtggagcaggtggaGCGCAGGCGGTTATCGTATTTTC	Insert tag after TfoS N929 F2
ABD643	TCGTTTTCTAAGGCTCGAATCGCC	Amplify native TfoS R2
**Primers for TfoR Constructs**		
**Primer**	**Sequence**	**Description**
BBC4983	GTGATACCACAAGCAACTATACC	TfoR F1
BBC4980	gcttaatcatgtttttaaggtgttgattttaACCTAAGCAAACATTGTTTTCG	Delete CBS in PtfoR R1
BBC4981	cgaaaacaatgtttgcttaggtTAAAATCAACACCTTAAAAACATGATTAAGC	Delete CBS in PtfoR F2
BBC4984	TCGTGCAACTTGAGAGTAACAAC	TfoR R2
VGP0478	tagaataggtactaatcagaatagtgaggagACCTAAGCAAACATTGTTTTCG	Insert 4X tetO in PtfoR R1
VGP0480	CTCCTCACTATTcTGATTAGTACC	Amplify 4X tetO F
BBC4338	CTCATTAGGCACtCCAGGC	Amplify 4X tetO R
VGP0479	gcctggagtgcctaatgagTAAAATCAACACCTTAAAAACATGATTAAGC	Insert 4X tetO in PtfoR F2
BBC706	CCACACATTATACGAGCCGATG	Amplify TfoR for Ptac R1

BBC708	CATCGGCTCGTATAATGTGTGGATTCCAGAGGTGGTATGA CC	Amplify TfoR for Ptac F2
